# C4b-binding protein inhibits particulate- and crystalline-induced NLRP3 inflammasome activation

**DOI:** 10.3389/fimmu.2023.1149822

**Published:** 2023-05-22

**Authors:** Damien Bierschenk, Nikolina Papac-Milicevic, Ian P. Bresch, Valentina Kovacic, Serena Bettoni, Mateusz Dziedzic, Rick A. Wetsel, Susanne Eschenburg, Christoph J. Binder, Anna M. Blom, Ben C. King

**Affiliations:** ^1^ Division of Medical Protein Chemistry, Department of Translational Medicine, Lund University, Malmö, Sweden; ^2^ Department of Laboratory Medicine, Medical University of Vienna, Vienna, Austria; ^3^ Institute for Biophysical Chemistry, Hannover Medical School, Hannover, Germany; ^4^ Cluster of Excellence RESIST (EXC 2155), Hannover Medical School, Hannover, Germany; ^5^ Research Center for Immunology and Autoimmune Diseases, Brown Foundation Institute of Molecular Medicine, McGovern Medical School, University of Texas Health Science Center at Houston, Houston, TX, United States

**Keywords:** inflammasome, C4BP, cytokine, pyroptosis, gout, MSU, silica

## Abstract

Dysregulated NLRP3 inflammasome activation drives a wide variety of diseases, while endogenous inhibition of this pathway is poorly characterised. The serum protein C4b-binding protein (C4BP) is a well-established inhibitor of complement with emerging functions as an endogenously expressed inhibitor of the NLRP3 inflammasome signalling pathway. Here, we identified that C4BP purified from human plasma is an inhibitor of crystalline- (monosodium urate, MSU) and particulate-induced (silica) NLRP3 inflammasome activation. Using a C4BP mutant panel, we identified that C4BP bound these particles *via* specific protein domains located on the C4BP α-chain. Plasma-purified C4BP was internalised into MSU- or silica-stimulated human primary macrophages, and inhibited MSU- or silica-induced inflammasome complex assembly and IL-1β cytokine secretion. While internalised C4BP in MSU or silica-stimulated human macrophages was in close proximity to the inflammasome adaptor protein ASC, C4BP had no direct effect on ASC polymerisation in *in vitro* assays. C4BP was also protective against MSU- and silica-induced lysosomal membrane damage. We further provide evidence for an anti-inflammatory function for C4BP *in vivo*, as *C4bp^-/-^* mice showed an elevated pro-inflammatory state following intraperitoneal delivery of MSU. Therefore, internalised C4BP is an inhibitor of crystal- or particle-induced inflammasome responses in human primary macrophages, while murine C4BP protects against an enhanced inflammatory state *in vivo*. Our data suggests C4BP has important functions in retaining tissue homeostasis in both human and mice as an endogenous serum inhibitor of particulate-stimulated inflammasome activation.

## Introduction

Specific intracellular pattern recognition receptors (PRRs), such as NOD-like receptors (NLRs), survey the intracellular milieu to initiate a rapid innate immune-driven response upon detection of invading pathogens and sterile danger-associated molecular patterns (DAMPs). Some PRRs are involved in formation of the inflammasome, an intracellular complex responsible for the production of the cytokines IL-1β and IL-18 (1). Of the different cytosolic PRRs known to trigger inflammasome formation, NLRP3 is the best studied. The NLRP3 inflammasome is activated in a two-step process. In the first ‘priming’ step, extracellular signals such as TLR ligands cause up-regulation of pro-IL-1β and inflammasome components (2), and in the second, triggering of cytosolic PRRs such as NOD-like receptor protein 3 (NLRP3) leads to formation of the inflammasome complex, with oligomerisation of the apoptosis-associated speck-like protein containing a caspase recruitment domain (ASC), and recruitment and activation of caspase-1, which cleaves pro-IL-1β and IL-18 into the active cytokines, and cleaves gasdermin D to drive pyroptosis, a highly inflammatory form of cell death (3). A wide variety of NLRP3 inflammasome-activating stimuli have been identified, including various pathogens, host-derived molecules (e.g. ATP, monosodium urate crystals [MSU] and amyloid proteins) and insoluble particles (e.g. silica dioxide [SiO_2_] and asbestos) (4). While inflammasomes are an essential component of the innate immune system, inappropriate or chronic activation of this cascade can lead to disease (5). Silicosis and gout are two diseases which are, respectively, driven by a SiO_2_- or MSU-induced sterile inflammation. SiO_2_ and MSU are well-established activators of the NLRP3 inflammasome pathway (6, 7). Resultant release of IL-1β causes signalling *via* the IL-1 receptor, which is expressed by a wide-variety of cell types and drives inflammation at a local and systemic level. For example, mature IL-1β is described as the main driver of the pro-inflammatory environment in gout-affected joints (8), or in lungs of silicosis patients (9). Indeed, IL-1β neutralisation, or limiting generation of mature IL-1β, reduced SiO_2_-induced inflammation in mice (10) and gouty arthritis attacks in human (11, 12).

Previously we have established that C4b-binding protein (C4BP), a complement inhibitor found in serum, inhibits islet amyloid poly peptide (IAPP)-induced NLRP3 inflammasome activation (13). IAPP is one of several disease-causing amyloids or misfolded proteins that activate the NLRP3 inflammasome, as for example amyloid β- and superoxide dismutase 1-induced inflammasome activation contribute to development of Alzheimer’s disease or amyotrophic lateral sclerosis, respectively (14). IAPP oligomers trigger inflammasome activation in pancreatic islets, which contributes to type 2 diabetes pathogenesis (15). C4BP directly binds IAPP, preventing amyloid fibrillation and fibrillation-associated lysosomal membrane damage after uptake by macrophages, which resulted in reduced IL-1β secretion from the monocytic THP-1 cell line and primary human monocyte-derived macrophages (HMDM). C4BP also inhibited IL-1β secretion from THP-1 cells after stimulation with SiO_2_- or MSU (13), which also cause NLRP3 activation *via* induction of lysosomal damage after uptake. The main source of circulating serum C4BP is the liver (16), however C4BP expression is not limited to this organ. C4BP is also expressed in the lung (17), the site of silica pathogenesis, and its expression can be elevated at sites relevant to inflammasome-driven diseases, such as the joint of rheumatoid arthritis patients (18) or the brain of Alzheimer patients (19). Despite the high or elevated C4BP expression at sites relevant to silicosis and gout it is currently unclear how C4BP inhibits SiO_2_- or MSU-induced inflammasome activation. Here, we investigated whether C4BP directly binds such molecules, and how C4BP affects MSU and SiO_2_-induced inflammasome activation in HMDM and mice. We show that in the absence of serum, C4BP binds MSU and SiO_2*via*_ specific protein domains on the C4BP α-chain, and protects against lysosomal membrane damage after uptake in HMDM. We also demonstrate that C4BP retains tissue homeostasis in MSU-injected mice. Therefore, our studies highlight new complement-independent C4BP-mediated inflammasome inhibitory functions in both human and mouse, suggesting broader implications in maintaining tissue homeostasis than established previously.

## Materials and methods

### Proteins

Plasma C4BP, recombinant C4BP and C4BP complement control protein (CCP)-domain deletion mutants were purified as described previously (20, 21), with the minor modification that eluted proteins were extensively dialysed against phosphate-buffered saline. Decay-accelerating factor (DAF) was expressed as Fc-dimer, and purified by affinity chromatography using protein A-Sepharose followed by anion-exchange chromatography, as described previously (22).

### Cells

All experiments using human primary cells were approved under ethical permit number Dnr.2017/582. Human CD14^+^ monocytes were isolated from buffy coats, provided by the Region Skåne Blodcentralen, using density gradient centrifugation over LymphoPrep (Axis Shield, United Kingdom) followed by CD14+ magnetic-activated cell sorting (Miltenyi Biotec, Cologne, Germany). Isolated CD14^+^ monocytes were differentiated into human primary macrophages by culture for 7 days in Macrophage Serum-Free medium (Gibco, Waltham, MA, USA), supplemented with 50 ng/ml human M-CSF-1 (Peprotech, Rocky Hill, NJ, USA), 100 U/ml penicillin and 100 μg/ml streptomycin, prior to experimental procedures.

### Inflammasome activation assays

HMDM were primed for 4h with 100 ng/ml *Salmonella typhi* LPS (Sigma Aldrich, St. Louis, MO, USA) prior to changing the culture medium for Opti-MEM (Gibco) containing PBS, 100 μg/ml BSA or 100 μg/ml C4BP. Inflammasomes were then activated by addition of 10 μM nigericin (Sigma Aldrich), 200 μg/ml MSU (Sigma Aldrich) or 20 μg/ml SiO_2_ (Invivogen, San Diego, CA, USA). Inflammasome-induced cell death was monitored by lactate dehydrogenase (LDH) release measurement using the CytoTox 96 Non-Radioactive Cytotoxicity Assay (Promega, Madison, WI, USA). Cytokine secretion was measured using the Human IL-1β ELISA Basic kit (Mabtech, Nacka Strand, Sweden) or Human Total IL-18 DuoSet ELISA (R&D Systems, Minneapolis, MN, USA), according to manufacturer’s instructions. C4BP internalisation by HMDM following inflammasome activation was determined as follows; at 2h post-treatment HMDM were washed three times with PBS and subsequently lysed in lysis buffer (1.3% SDS, 44 mM TRIS-HCl, 10 mM DTT in 1x Laemmli buffer) prior to SDS-PAGE for immunoblot. The polymeric ASC speck was isolated as follows; Four hours post-stimulation cells were washed with 50 mM HEPES before lysis in 50 mM HEPES + 0.5% Triton X-100 + HALT protease and phosphatase inhibitor (Thermo Fisher Scientific, Waltham, MA, USA). Triton-insoluble polymeric ASC was then pelleted at 6000 g by 15-minute centrifugation at room temperature. Supernatants containing monomeric ASC were methanol/chloroform precipitated. Both fractions were lysed in lysis buffer prior to SDS-PAGE for immunoblot. Cells were treated for the duration of the experiment with the caspase-1/caspase-4 inhibitor VX-765 (50 μM; SelleckChem, Houston, TX, USA) to block pyroptosis.

### Immunoblot

Cell lysates and methanol-chloroform precipitated cell-free supernatants were separated using 4-20% Mini-Protean TGX Precast Protein Gels (Bio-Rad, Hercules, CA, USA), and transferred onto PVDF membranes (Bio-Rad). Membranes were blocked with fish gelatine (Norland products, Cranbury, NJ, USA) and then blotted with goat anti-IL-1β (R&D Systems AF-201-NA, 1:1000), rabbit anti-C4BP (Agrisera custom made, 1:5000), rabbit anti-tubulin (Abcam, Cambridge, UK; ab6046, 1:5000) or mouse anti-ASC (Santa Cruz, Dallas, TX, USA; B-3, 1:1000). Immunoblots were developed with appropriate horse radish peroxidase (HRP)-conjugated secondary antibodies (DAKO, Glostrup, Denmark; 1:5000), and subsequently developed using Immobilon ECL (Millipore, Darmstadt, Germany). Immunoblot targets from the same sample were detected on the same membrane, which was either cut and targets immunoblotted in parallel, or stripped and re-probed. Stripping of HRP activity was achieved with 30% H_2_O_2_ for 15 minutes at room temperature, prior to re-incubation with a subsequent primary antibody.

### Real-time PCR

RNA was isolated from HMDM using the RNeasy Plus Mini Kit (Qiagen, Hilden, Germany) according to manufacturer’s instructions. Isolated RNA was reverse transcribed into cDNA using Superscript IV reverse transcriptase (ThermoFisher). Gene expression was subsequently determined, with TaqMan Gene Expression Assays Hs01555413_m1 (*IL1β*) and Hs99999909_m1 (*HPRT*), using the ViiA 7 Real-Time PCR system (Applied Biosystems, Waltham, MA, USA)

### C4BP binding to MSU or silica

Plasma-purified C4BP, recombinant C4BP and CCP domain mutants of C4BP were fluorescently labelled using the Alexa Fluor 647 (AF647) microscale protein labelling kit (Invitrogen, Carlsbad, CA, USA). Successful protein labelling was confirmed using the Typhoon FLA 9000 Fluorescence imaging system after conventional SDS-PAGE, and protein concentrations post-labelling were determined using a previously published in-house C4BP ELISA (23). ELISA results were used to account for differences in protein concentration post-AF647 labelling, absolute volumes used in the binding assay to dilute proteins to 100 nM were as follows: plasma-purified C4BP 12.36 μl, recombinant C4BP 13.04 μl, ΔCCP1 9.28 μl, ΔCCP2 9.88 μl, ΔCCP3 11.80 μl, ΔCCP4 8.08 μl, ΔCCP5 9.92 μl, ΔCCP6 39.04 μl, ΔCCP7 9.76 μl, ΔCCP8 22.52 μl, PBS was added to obtain a total volume of 50 μl. AF647-labeled proteins were then serially diluted, in PBS, to 25 nM prior to addition of an equal volume of 100 μg MSU or 10 μg SiO_2_ resuspended in buffer 1 (1% BSA in PBS, supplemented with 2 mM EDTA). Proteins and activators were gently mixed by swirling and incubated for 20 minutes at room temperature in the dark. Mixtures were centrifuged for 3 minutes at 300 g and unbound protein was aspirated. Pelleted MSU and SiO_2_ were gently resuspended in buffer 1 by manual pipetting, and samples were run on the Beckman Coulter CytoFLEX flow cytometer. AF647 mean fluorescence intensity was then determined for each sample using FlowJo software (Tree Star, Ashland, OR, USA).

### Proximity ligation assay

HMDM were plated in 12 well chambers (Ibidi, Martinsried, Germany) prior to stimulation with 100 ng/ml LPS to upregulate inflammasome component expression. Cell culture medium was exchanged for Opti-MEM containing 100 μg/ml BSA or 100 μg/ml plasma-purified C4BP supplemented with 10 μM nigericin, 200 μg/ml MSU or 20 μg/ml SiO2. VX-765 (50 μM) was added to all samples to prevent pyroptosis. Four hours post-treatment HMDM were washed with PBS and fixed using 4% PFA prior to the PLA. In short, 50 mM NH4CL was used to quench remaining aldehyde prior to blocking and membrane permeabilisation with 3% BSA and 0.1% Triton X-100 in PBS. Permeabilised HMDM were incubated with mouse anti-ASC (Santa Cruz B-3, 1:1200) and rabbit anti-C4BP (Agrisera custom made, 1:500), diluted in 3% BSA and 0.1% Triton X-100, for 1 hour at 37°C. PLA was then performed using the Duolink *In Situ* Detection Reagents Orange (Sigma Aldrich) according to manufacturer recommendations with the Duolink *In Situ* PLA Probe anti-mouse MINUS (Sigma Aldrich) and Duolink *In Situ* PLA Probe anti-rabbit PLUS (Sigma Aldrich). The PLA reaction was allowed to continue for 100 minutes, prior to mounting of the cells using the Duolink *In Situ* Mounting Medium containing DAPI (Sigma Aldrich). IgG control antibodies were of the same antibody subtype, Normal Rabbit IgG (Merck Millipore, 12-370) and mouse IgG1, κ (Biolegend, San Diego, CA, USA, 401401), at the same concentration as primary antibodies used in the PLA.

### Lysosomal galectin-3 staining

HMDM were plated in 12 well chambers (Ibidi) prior to stimulation with 100 ng/ml LPS to upregulate inflammasome component expression. Culture medium was exchanged for Opti-MEM containing 100 μg/ml BSA or 100 μg/ml plasma-purified C4BP supplemented with 200 μg/ml MSU, 50 μg/ml SiO_2_ or 2 mM L-Leucyl-L-Leucine methyl ester (LLoMe, Cayman Chemicals, Ann Arbor, MI, USA). VX-765 (50 μM) was added to all samples to prevent pyroptosis. Two- or 4-hours post-treatment cells were washed with PBS, and cells were fixed using 4% PFA. Fixed cells were stained for galectin-3 and nuclei, using DAPI, according to established protocols (24). In short, 50 mM NH_4_Cl was used to quench remaining aldehyde prior to blocking and membrane permeabilisation with 1% BSA, 5% normal Donkey serum and 0.3% Triton X-100 in PBS. Mouse anti-human galectin-3 antibody (BD Biosciences, San Jose, CA, USA; B2C10 1:50) was added to permeabilised cells and incubated overnight at 4°C. After washes, samples were incubated with AF488-labelled goat anti-mouse IgG (Invitrogen, Waltham, MA) for 1 hour at 37°C, prior to mounting with DAPI-containing mounting medium (Sigma Aldrich). Samples were imaged using the LSM800 confocal microscope (Carl Zeiss Gmbh, Jena, Germany) and analysed using Zen Black software (Carl Zeiss Gmbh).

### Macrophage influx after intraperitoneal MSU injection

All experimental protocols using mice were approved by the institutional animal experimentation committee of the Medical University of Vienna and the Austrian Ministry of Science. *C4bp* ^-/-^ mice were backcrossed to the C57BL/6J background, and all experiments were performed with age and gender-matched control mice, all animals were between 8-10 weeks of age. C4BP deficient animals were originally generated by Rick A. Wetsel (25) at The University of Texas, TX, U.S.A. MSU was injected into the mouse peritoneum at 0.08 mg/g bodyweight in sterile PBS, and 5 hr or 8hr post-injection mice were sacrificed by cervical dislocation and peritoneal lavage was performed using 5ml of sterile Hank’s Balanced Salt solution. Cells within the peritoneal lavage were counted using a CASY cell counter, and afterwards collected by centrifugation for 10 min at 400*g* and resuspended in PBS + 1% BSA to prepare for flow cytometric analysis. 1x10^6^ of cells in a V-bottom plates were treated with 100µl of 2.5 μg/ml anti-CD16/32 antibody (Clone 93, Thermo Fisher Scientific) for 20 minutes at 4°C to block the Fcγ receptors. After washing in PBS + 1% BSA buffer cells were then stained with antibody cocktail containing; anti-CD11b APC (clone M1/70, Thermo Fisher Scientific), anti-Ly6C FITC (clone HK1.4, BioLegend) and anti-Ly6G PE (clone 1A8, BioLegend) or anti-CD11b FITC (clone M1/70, Biolegend) and anti-F4/80 APC (clone BM8, BioLegend). For the 8 hr analysis, cells were treated with a cocktail of anti-CD11b eFluor450 (48-0454-82, Thermo Fisher Scientific), anti-Ly6C BV605 (clone HK1.4, BioLegend, San Diego, CA, USA) and anti-Ly6G Alexa Fluor 647(clone 1A8, BioLegend) or anti-CD11b FITC (clone M1/70, Biolegend) and anti-F4/80 APC-Cy7 (clone BM8, BioLegend)) and anti-CD3e PE (145-2C11, eBiosciences), and anti-B220 PercP-Cy5.5 (RA3-6B2, eBioscience) for 20 minutes at 4°C. Fluorescence was measured using a Becton Dickinson FACSCalibur (5 hr) or a LSRFortessa™ (8 hr) (BD Bioscience), and data were analysed using FlowJo software.

### Microscale Thermophoresis

MST was performed according to established protocols (26), with minor modifications. The buffer was changed to 1x PBS and glutathione was added to a final concentration of 13 mM to mimic intracellular conditions. In short, a dilution series was prepared with unlabelled ASC^PYD^ and mixed with Atto488-labeled ASC^PYD^, TEV Protease to start polymerisation, and either plasma-purified or recombinant C4BP in their respective concentrations. The mixture was filled into capillaries, incubated and measured with 60% MST-power using the Monolith NT.115Pico system (NanoTemper Technologies, Munich, Germany) under control of NT Software (version 2.2.1).

### Statistical analysis

Data were tested for significance using a paired parametric student’s *t* test, one-way ANOVA with Dunnett’s multiple comparison test, or two-way ANOVA with Sidak’s multiple comparison test or Tukey’s multiple comparison test, where indicated, using GraphPad Prism 7. Data were deemed statistically significant when *p* < 0.05(*), *p* < 0.01(**), *p* < 0.001(***) or *p* < 0.0001(****)

## Results

### C4BP binds SiO_2_ and MSU and inhibits the inflammasome in HMDM

**Figure 1:**
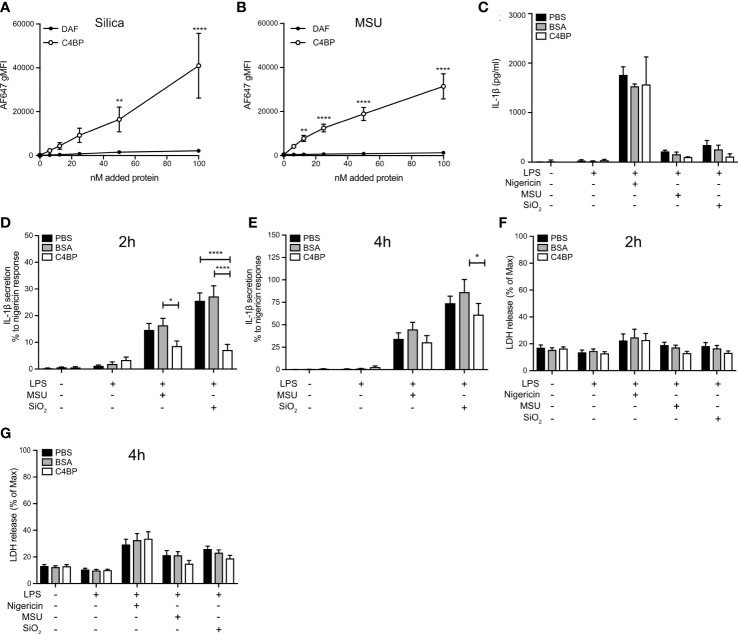
C4BP binds SiO_2_ and MSU and inhibits inflammasome signalling. **(A, B)** Flowcytometric analysis of Alexa Fluor 647-labeled plasma-purified C4BP or DAF binding to Silica and MSU at various concentrations. **(C–G)** HMDM were primed 4h with LPS prior to treatment with PBS, BSA or plasma-purified C4BP and activation by indicated inflammasome activators. **(C–E)** Cell supernatants were analysed for IL-1β secretion using ELISA 2h **(C, D)** and 4h **(E)** post-treatment. Human donors showed a wide-variety in absolute secreted IL-1β secretion values, therefore **(C)** is a representative of the IL-1β secretion profile triggered by the various activators, while values in **(D)** and **(E)** were converted to percentages relative to the maximum response (nigericin). **(F, G)** Cytotoxicity was assayed by cell supernatants analysis for lactate dehydrogenase release at 2h **(F)** and 4h **(G)** post-treatment. C4BP binding graphs are mean + SEM of five independent experiments (**A, B**). Bar graphs are mean + SD of duplicate well stimulations of one human donor **(C)** or mean + SEM of data pooled from 6 independent human donors **(D–G)**. Data were tested for significance by two-way ANOVA using either Sidak’s multiple comparison test **(A, B)** or the Tukey’s multiple comparison test **(D–G)**. *p < 0.05, **p < 0.01 ****p < 0.0001. gMFI, geometric mean fluorescence intensity.

Given that C4BP inhibited IAPP-, SiO_2_-, and MSU-induced inflammasome responses in the THP-1 cell line, we hypothesised that C4BP also bound SiO_2_ particles and MSU crystals, in addition to IAPP (13). Indeed AF647-labeled C4BP purified from serum, but not DAF as a CCP domain-containing control protein, strongly bound SiO_2_ particles and MSU crystals as investigated by flow cytometry (**Figures 1A, B**). Next, we investigated whether C4BP also acts as an inhibitor of the NLRP3 inflammasome pathway in HMDM. Macrophages were exposed to nigericin, a soluble NLRP3 inflammasome activator, SiO_2_ particles or MSU crystals, in the presence of C4BP, or BSA as a control protein, and inflammasome responses were monitored. C4BP significantly decreased SiO_2_- and MSU-induced IL-1β secretion at 2h post-treatment, and SiO_2_-induced IL-1β secretion at 4h post-treatment, compared to the BSA-treated control cells (**Figures 1C–E**). Another hallmark of inflammasome activation is cell death (27), which we assayed using the LDH release assay. We found that C4BP did not significantly reduce SiO_2_- and MSU-induced LDH release at 2h or 4h post-treatment compared to BSA-treated cells (**Figures 1F, G**). In no experiments did C4BP affect nigericin-induced responses, suggesting that C4BP specifically inhibits NLRP3 signalling by insoluble activators, such as crystals or particles.

### C4BP does not inhibit HMDM priming

**Figure 2:**
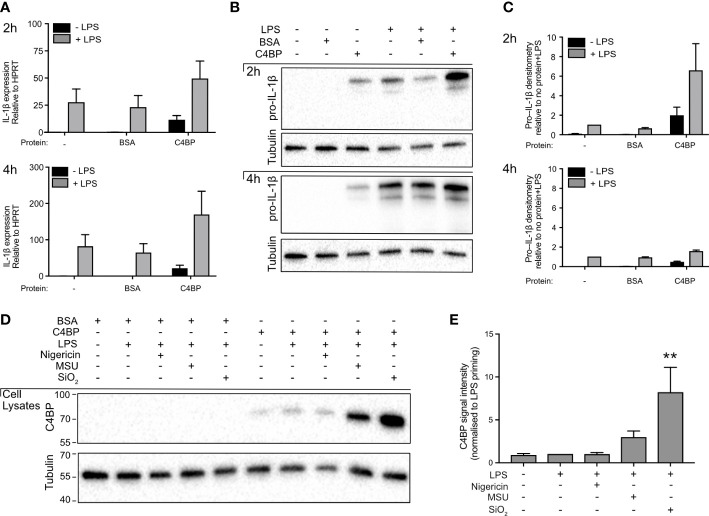
C4BP does not inhibit Pro-IL-1β expression and is internalised by HMDM. **(A, B)** HMDMs were treated with PBS, BSA or plasma-purified C4BP, with or without LPS co-stimulation, for 2h or 4h. Expression of *IL1B* was assayed by quantitative PCR **(A)**, and pro-IL-1β expression was analysed by western blot where tubulin served as loading control **(B)**. **(C)** Densitometry analysis for pro-IL-1β western blots, normalised to pro-IL-1β signal intensity in PBS-treated, LPS stimulated HMDM. **(D)** C4BP internalization was assayed by western blot at 2h post-treatment with the indicated inflammasome activator in 4h LPS-primed HMDM. **(E)** Densitometry analysis for C4BP western blot, normalised to C4BP signal intensity in LPS-primed HMDM. Western blots are a representative of data from 3 independent human donors **(B)** or from 5 independent human donors **(D)** used to perform densitometry analysis **(C, E)**.

Pro-IL-1β expression is low in unstimulated macrophages and therefore, prior to experimental inflammasome activation, macrophages are often stimulated with a Toll-like receptor (TLR) ligand (28). In our experiments, TLR stimulation was achieved using LPS, which signals *via* TLR4. We investigated whether HMDM treatment with C4BP affects pro-IL-1β expression, as the C4BP-mediated inhibition of IL-1β release could be explained by a reduction in priming and expression of pro-IL-1β, which is cleaved by caspase-1 into mature IL-1β upon inflammasome activation. C4BP had no significant effect on levels of *IL1β* mRNA in LPS-primed HMDM, (**Figure 2A**), and pro-IL-1β protein levels in cell lysates (**Figures 2B, C**) were not inhibited compared to LPS and PBS, or LPS and BSA control conditions, confirming that C4BP does not inhibit the priming step, as also seen previously (13). Some apparent increases in pro-IL-1β protein were seen in primed cells in the presence of C4BP, attributed to an inhibitory effect of C4BP on the processing of pro-IL-1β to the mature form.

### C4BP is internalised in crystal- or particle-stimulated HMDM

Given that C4BP presence did not negatively affect pro-IL-1β expression following extracellular TLR-stimulation, we hypothesised MSU- or SiO_2_-bound C4BP may inhibit NLRP3 activation intracellularly. We previously characterised that C4BP is co-internalised when bound to the inflammasome activator IAPP (13), as well as the rat (29). Internalisation of MSU and SiO_2_ by human macrophages is well characterised, thus we asked whether C4BP binding to MSU or SiO_2_ also promotes C4BP uptake in HMDM. We found that C4BP uptake was increased in MSU- or SiO_2-_stimulated HMDM, when compared to LPS priming alone. In nigericin-stimulated HMDM, C4BP was internalised at similar amounts as the LPS priming alone control (**Figures 2D, E**). These data indicate that cellular uptake of C4BP bound to particulate inflammasome activators may be crucial for C4BP-mediated inflammasome inhibition in MSU- or SiO_2_-stimulated HMDM, while HMDM priming responses remain unaffected by C4BP.

### C4BP inhibits particulate-mediated ASC oligomerisation

**Figure 3:**
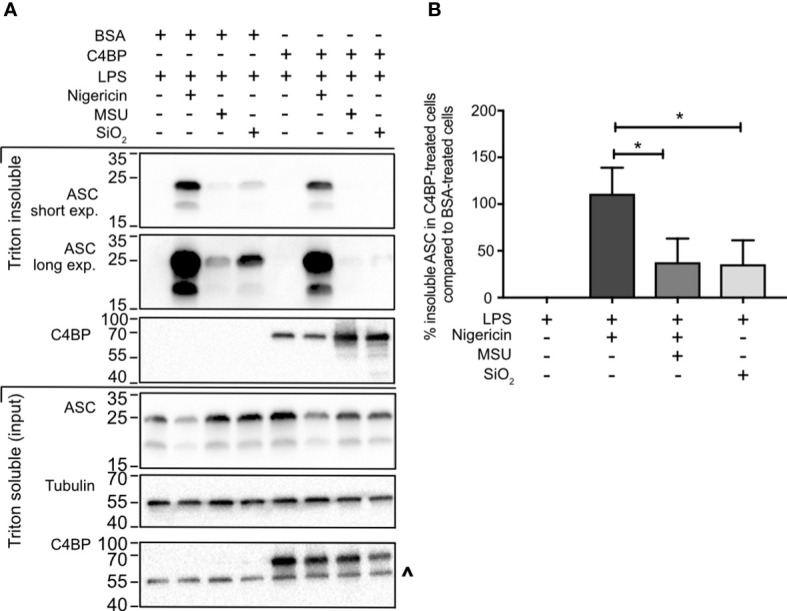
C4BP prevents ASC speck formation. **(A)** HMDM were LPS-primed for 4h prior to treatment with BSA or plasma-purified C4BP and 50 μM VX-765 to prevent cell death and subsequent dissociation of the ASC speck. Four hours post-treatment with the indicated inflammasome activator cells were lysed in Triton X-100-containing buffer to fractionate monomeric ASC (Triton X-100 soluble) and polymeric ASC (Triton X-100 insoluble). Triton X-100 soluble and insoluble fractions were analysed using western blot for ASC and C4BP while tubulin was used as a loading control. **(B)** ASC western blot signal strength in C4BP-treated samples compared to BSA-treated samples, as determined by densitometry analysis. Western blot **(A)** is a representative of data from 5 independent human donors used to perform the densitometry analysis **(B)**. Data were tested for significance by one-way ANOVA with the Dunnett’s multiple comparison test **(B)**. *p < 0.05. ^ indicates remaining tubulin signal after stripping membrane and reprobing for C4BP.

Most canonical inflammasomes recruit the inflammasome adaptor protein ASC, triggering its oligomerisation into a larger ‘speck’-like structure, which facilitates caspase-activation and downstream signalling (4). To investigate whether C4BP inhibits ASC speck formation, we took advantage of the differences in Triton solubility between monomeric ASC (triton soluble) and polymeric ASC (triton insoluble) (30). To assay whether C4BP inhibits ASC speck formation, we compared ASC immunoblot intensity in the triton insoluble fraction between BSA- or C4BP-treated inflammasome-stimulated HMDM. Cell death and extracellular ASC speck release were prevented by HMDM treatment with the caspase-1/-4 inhibitor VX-765. In concordance with the reduction in cytokine secretion, C4BP significantly inhibited SiO_2_- and MSU-induced ASC speck formation, with a significant decrease in proportions of Triton-insoluble ASC (**Figures 3A, B**), while no differences were observed in nigericin-induced ASC speck formation. Our results indicate that plasma-purified C4BP is an inhibitor of crystalline- or particle-induced NLRP3 inflammasome activation, acting upstream of ASC speck formation, while soluble NLRP3 agonist-induced responses remain unaffected.

### The C4BP α-chain mediates inflammasome inhibition in human macrophages

**Figure 4:**
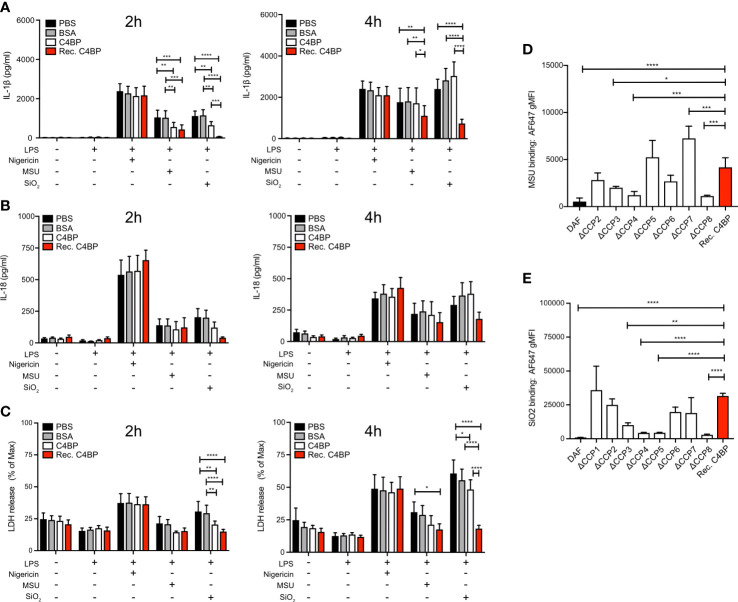
The C4BP α-chain mediates inflammasome inhibition and binding to silica and MSU. **(A–C)** HMDM were LPS-primed for 4h prior to treatment with PBS, BSA, plasma-purified C4BP or recombinant C4BP and subsequent stimulation with the indicated inflammasome activator. Cell culture supernatants were analysed for secretion of IL-1β **(A)** or IL-18 **(B)** and for LDH release **(C)** at 2h and 4h post-treatment. **(D, E)** Flowcytometric analysis of Alexa Fluor 647-labeled recombinant C4BP (Rec. C4BP), DAF, or specific C4BP CCP-deletion mutants, binding to MSU **(D)** or SiO_2_ **(E)**. Data were tested for significance by two-way ANOVA using the Tukey’s multiple comparison test **(A–C)** or by one-way ANOVA with the Dunnett’s multiple comparison test **(D, E)**. Bar graphs are mean + SEM of data pooled from 7 independent human donors **(A–C)**, or pooled from 4 independent experiments (**D, E**). *p< 0.05, **p< 0.01, ***p< 0.001, ****p< 0.0001. gMFI, geometric mean fluorescence intensity.

Approximately 80% of C4BP in human plasma is an isoform that consists of 7 α-chains and 1 β-chain. Each α-chain consists of 8 CCP domains, while every β-chain consists of 3 CCP domains (17). In circulation, the C4BP β-chain binds to the vitamin K-dependent protein S (PS) (31), an interaction utilised to purify the C4BP-PS complex used thus far in this study. Therefore, we next asked the question whether the C4BP-mediated inflammasome inhibition may be due to enhanced concentrations of PS, or the presence of C4BP-PS complexes. We expressed and purified recombinant C4BP, which lacks the β-chain (21), and investigated whether HMDM NLRP3 inflammasome signalling was inhibited in the absence of β-chain/PS. Indeed, recombinant C4BP significantly inhibited MSU- and SiO_2_-induced IL-1β secretion at 2h and 4h post-treatment (**Figure 4A**). When we analysed IL-18 secretion we found that there was tendency, which did not reach statistical significance, towards recombinant C4BP inhibition of MSU- and SiO_2_-induced IL-18 secretion (**Figure 4B**). Recombinant C4BP significantly inhibited SiO_2_-induced cytotoxicity at both time points, while MSU-induced cytotoxicity was not significantly reduced (**Figure 4C**). Interestingly, inhibition of MSU- and SiO_2_-induced inflammasome activation was more potent and long-lasting in recombinant C4BP-treated HMDM, as compared to plasma-purified C4BP-treated HMDM. These data suggested that the C4BP β-chain, and thus PS, is dispensable for C4BP-mediated inflammasome inhibition, and that the C4BP α-chain is required for inflammasome inhibition. To identify which C4BP α-chain CCP domains were required for the inhibition we recombinantly expressed and purified a C4BP mutant panel in which each mutant lacks a specific α-chain CCP domain (21). As expected, recombinant WT C4BP bound MSU and SiO_2_, while DAF used as negative control showed no binding. C4BP mutants lacking CCP3, CCP4 or CCP8 were unable to bind MSU (**Figure 4D**), while CCP4, CCP5 and CCP8 were crucial for binding to SiO_2_ (**Figure 4E**). Deletion of CCP1 resulted in a severely enhanced binding to MSU (**Supplementary Figure 1**), and the ΔCCP1 mutant has therefore been omitted from the data set in **Figure 4D**. Interestingly, the ΔCCP3 mutant showed significantly reduced, but not abolished, binding towards SiO_2_, suggesting C4BP binding to silica is augmented by the CCP3 domain (**Figure 4E**). Collectively, these data indicate that after binding crystalline or particulate inflammasome activators, C4BP α-chain internalisation mediates inflammasome inhibition in human macrophages.

### C4BP does not directly affect ASC polymerisation

**Figure 5:**
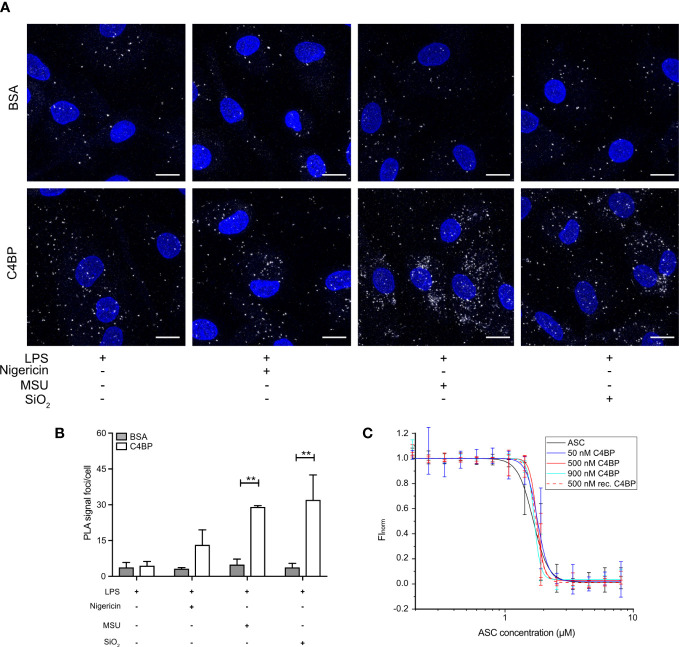
Internalised C4BP is in proximity with the inflammasome complex. **(A, B)** HMDM were LPS-primed for 4h prior to treatment with BSA or plasma-purified C4BP, 50 μM VX-765 to prevent cell death, and the indicated inflammasome activator for 2h. **(A)** Confocal microscopy z-stack maximum projection images of a proximity ligation assay (PLA) between C4BP and ASC. PLA signal foci are represented in white, nuclei in blue. **(B)** Determination of C4BP-ASC PLA signal foci/cell in inflammasome-stimulated HMDM. **(C)** Microscale thermophoresis of Atto488-labeled ASC^PYD^ with or without plasma-purified C4BP or recombinant C4BP at indicated concentrations. Confocal microscopy images are a representative of data from 3 independent human donors **(A)**. Data were tested for significance by two-way ANOVA using the Sidak’s multiple comparison test **(B)**. Bar graphs are mean + SEM of data pooled from 3 independent human donors **(B)**. **p < 0.01. Scale bars, 10 μm.

ASC polymerisation is a crucial step in canonical inflammasome signalling, as it provides the caspase-1 activation hub required for downstream signalling. ASC polymerisation has many similarities to amyloid fibrillation, and ASC specks can even cross-seed amyloid formation (32). As C4BP directly prevents IAPP amyloid fibrillation (11), and we established that C4BP internalisation into particulate-stimulated HMDM inhibited ASC speck formation (**Figures 3A, B**), we hypothesised that C4BP may also directly interact with ASC to prevent its polymerisation. To investigate whether C4BP co-localises with ASC upon NLRP3 inflammasome stimulation, we performed a proximity ligation assay (PLA) to identify co-localisation between C4BP and ASC. PLA was performed on nigericin-, MSU- or silica-stimulated HMDM that were treated with C4BP, or BSA as a negative control for PLA signal. As expected, BSA-treated HMDM showed very few PLA foci/cell, independent of the inflammasome activator present. PLA foci/cell were significantly increased in C4BP-treated HMDM that were stimulated with MSU or silica, but not in cells stimulated with nigericin (**Figures 5A, B**). As expected, when primary antibodies were substituted for IgG control antibodies, the PLA signal foci/cell were dramatically reduced (**Supplementary Figure 2**), confirming specificity. These data suggest that MSU or silica-bound C4BP co-localises with the inflammasome adaptor protein ASC after uptake, but only upon C4BP internalisation together with the activator. Intracellular C4BP concentrations are lower in nigericin-stimulated HMDM (**Figure 2D**), providing further evidence that binding to a particulate activator, followed by uptake, is required for the inflammasome inhibitory effect of C4BP.

We next investigated whether C4BP can directly prevent ASC polymerisation *in vitro* using an extensively validated Microscale Thermophoresis (MST) approach. We have demonstrated that ASC concentrations required for *in vitro* initiation of polymerisation are increased in the presence of inhibitory factors (e.g. increased KCl concentrations or reduced pH values) (26). We hypothesised that ASC polymerisation kinetics may be similarly affected in the presence of C4BP. MST detects alterations in fluorescently labelled molecule movement along a temperature gradient. As thermophoretic behaviour of molecules is affected by size, charge and shell hydration, this method provides an excellent platform to detect the increase in complex size upon ASC polymerisation. ASC is a bi-partite protein that consists of a PYD and CARD domain, of which the ASC^PYD^ is required for ASC speck filament formation upon inflammasome activation (33). Atto 488-labelled ASC^PYD^ was allowed to polymerise in the absence of C4BP, or in the presence of plasma-purified C4BP or recombinant C4BP, to investigate C4BP-mediated changes in thermophoretic behaviour. Increased concentrations of plasma-purified or recombinant C4BP in the assay buffer did not significantly affect the concentration of ASC^PYD^ required for polymerisation (**Figure 5C**). Thus, combined, our data indicates that C4BP is internalised upon HMDM stimulation with MSU or silica, where it subsequently co-localises with ASC, but does not directly interfere with ASC^PYD^ polymerisation.

### C4BP prevents MSU-induced lysosomal membrane damage

**Figure 6:**
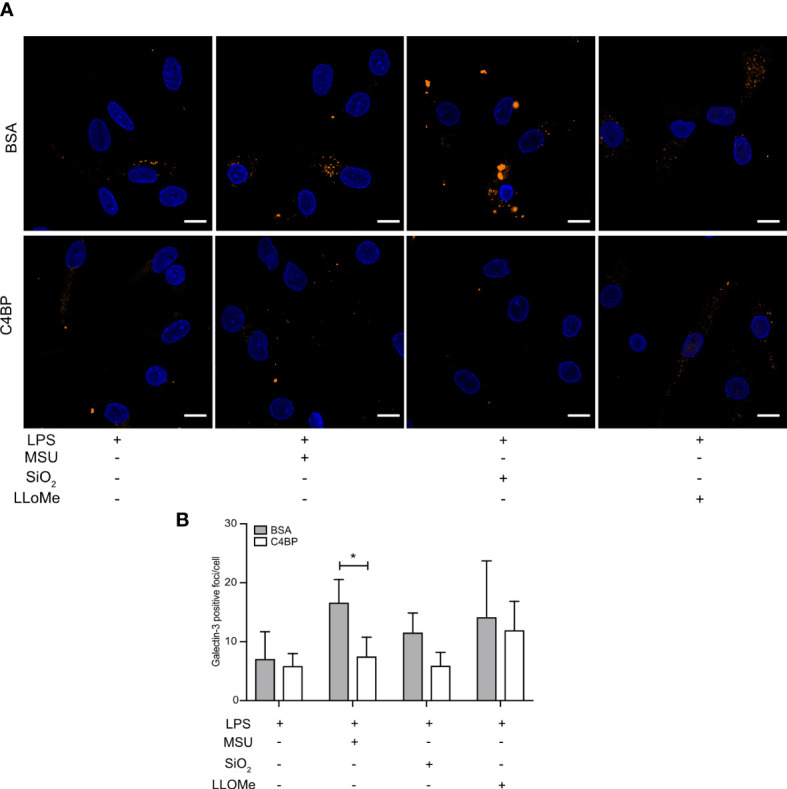
C4BP protects against MSU-induced lysosomal membrane damage. **(A, B)** HMDM were LPS-primed for 4h, prior to treatment with BSA or plasma-purified C4BP, 50 μM VX-765 to prevent cell death, and the indicated inflammasome activator for 2h or 4h or lysosomal membrane-damaging agent LLoMe for 1h. **(A)** Confocal microscopy images of damaged lysosome-associated galectin-3 puncta. **(B)** Determination of galectin-3 puncta/cell in untreated, inflammasome- or LLoMe-stimulated HMDM. Confocal images are representative of data from five independent human donors **(A)**. Bar graphs are mean + SEM of data pooled from 5 independent human donors **(B)**. Scale bars, 10 μm. *p < 0.05 by 2-way ANOVA.

SiO_2_ particles and MSU crystals are well-established lysosomal membrane damaging agents (34, 35). As C4BP inhibits IAPP fibrillation and IAPP-induced lysosomal membrane damage (13), which is dependent on active fibril formation (36), we investigated whether C4BP was also protective against lysosomal membrane damage induced by non-fibrillating SiO_2_ particles or MSU crystals in HMDM. The galectin puncta formation assay is a highly sensitive assay to assess lysosomal membrane damage, in which cytosolic galectins (e.g. galectin-1, -3 and -9) accumulate in puncta at the site of lysosomal membrane damage (37). The galectin-3 puncta formation assay was used to investigate whether C4BP is protective against lysosomal membrane damage in LPS priming-, SiO_2_- or MSU-stimulated HMDM, in which LPS priming stimulation served as a negative control for lysosomal membrane damage. LLoMe is widely established to trigger lysosomal membrane damage in a cathepsin C-dependent manner, and served as a positive control (38). As expected, LPS-primed HMDM mainly showed uniform galectin-3 staining, whereas clear galectin-3 puncta were observed in LLoMe-treated HMDM. We observed significantly more MSU-induced galectin-3 puncta in BSA-treated HMDM as compared to C4BP-treated HMDM, and while SiO_2_-induced galectin-3 puncta were not significantly decreased in C4BP-treated HMDM there was a clear tendency towards C4BP being protective against SiO_2_-induced lysosomal membrane damage (**Figures 6A, B**). Therefore, these data indicate that C4BP is protective against particulate-induced lysosomal membrane damage. Therefore, our data suggest that C4BP not only prevents fibrillation-induced lysosomal damage but is also protective against lysosomal membrane damage induced by larger non-fibrillating crystals or particles.

### Murine C4BP limits inflammation *in vivo*

**Figure 7:**
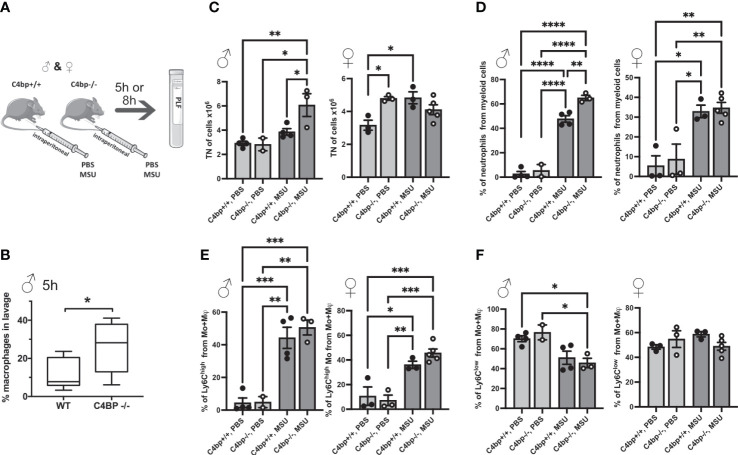
Murine C4BP protects against inflammation *in vivo*. **(A)** WT and *C4bp* ^-/-^ mice were injected intraperitoneally with MSU, and after 5h or 8h, immune cell influx into the peritoneal cavity was analysed by flow cytometric analysis of peritoneal lavage fluid. **(B)** Macrophage influx into the peritoneal cavity was assessed at 5h in male mice. At 8h, total cell number **(C)**, or proportion of neutrophils **(D)**, Ly6C^high^ monocytes **(E)** or Ly6C^low^ monocytes **(F)** were assessed in male mice (left panels) and female mice (right panels). Statistical significance was tested using a parametric student’s *t* test **(B)**, or one-way ANOVA with Tukey’s post test **(C–F)**. *p< 0.05, **p< 0.01, ***p< 0.001, ****p< 0.0001. Individual spots are individual mice, n = 3 – 5 per group.

We next sought to determine whether C4BP is an inflammasome inhibitor *in vivo.* Similar to human, C4BP is found at high concentrations in serum of male mice (160 μg/ml), although it is about 2.5x lower in female mice (60 μg/ml), due to influence of testosterone on expression of the gene (39, 40). To investigate protective functions of C4BP *in vivo*, we utilised an established model of inflammasome-driven sterile peritonitis (7) in which C57Bl/6J WT or *C4bp^-^* ^/-^ mice were injected intraperitoneally with MSU crystals and the influx of pro-inflammatory cells into the peritoneal cavity was monitored. In untreated control mice, levels of peritoneal cell populations were not significantly different between WT or *C4bp* ^-/-^ male or female mice (**Supplementary Figure 3**), showing that lack of C4BP does not affect levels of resident cells. Neutrophils made up less than 0.4% of retrieved cells in untreated mice. We then injected MSU into the peritoneal cavity and analysed results at 5 or 8 hrs (**Figure 7A**). At 5 hrs, we found a significant increase in pro-inflammatory macrophage influx in male *C4bp* ^-/-^ mice as compared to WT mice, suggesting that upon peritoneal MSU delivery, absence of C4BP leads to an enhanced pro-inflammatory state (**Figure 7B**). We also separately analysed larger numbers of mice at 8 hrs post-injection, including both males and females. At 8 hrs, there was a much larger influx of total cells (7C, left panel) and neutrophils (7D, left panel) in MSU-treated *C4bp* ^-/-^ males, compared to WTs, demonstrating a heightened proinflammatory response in the absence of C4BP. Surprisingly, there was no significant difference between MSU-treated WT and *C4bp* ^-/-^ females (7C, D, right panels), attributed to the lower levels of C4BP found in female mouse serum (39, 40). In both MSU-treated females and males, lack of C4BP led to a non-significant trend in increase of the influx of pro-inflammatory Ly6C^high^ monocytes at 8 hrs (7E), while there was no change in levels of Ly6C^low^ monocytes (7F).

## Discussion

We have identified that human C4BP is an inhibitor of SiO_2_- or MSU-induced NLRP3 inflammasome activation in HMDM. C4BP was originally identified as an inhibitor of the classical and lectin complement cascades. The complement system consists of more than 40 proteins, of which virtually all have reported genetic deficiencies in human (41). While in rare patient cases reduced C4BP protein levels were reported (42), full C4BP deficiency has thus far not been described (17), which strengthens our hypothesis that C4BP may have broader functions in maintaining tissue homeostasis. In addition to our previous findings that C4BP inhibits IAPP-induced NLRP3 activation, we have now identified C4BP as an inhibitor of a wider range of NLRP3 inflammasome activators. PLA suggested that, once internalised, C4BP co-localises with the inflammasome adaptor ASC. However, our MST experiments indicated that C4BP does not directly affect ASC^PYD^ polymerisation, suggesting C4BP acts *via* a different mechanism than other identified endogenous inflammasome signalling modulators, for example members of the CARD-only protein (COP) and PYD-only protein (POP) families that are reported to interfere with ASC binding to inflammasome complex components (43–45). While inflammasome adaptor proteins, such as NLRP3, induce ASC filament polymerisation *via* the ASC^PYD^ domain, we can currently not rule out that C4BP may interfere with ASC-dependent signalling due to interactions with the ASC^CARD^ domain, which is required for filament crosslinking (46).

**Figure 8:**
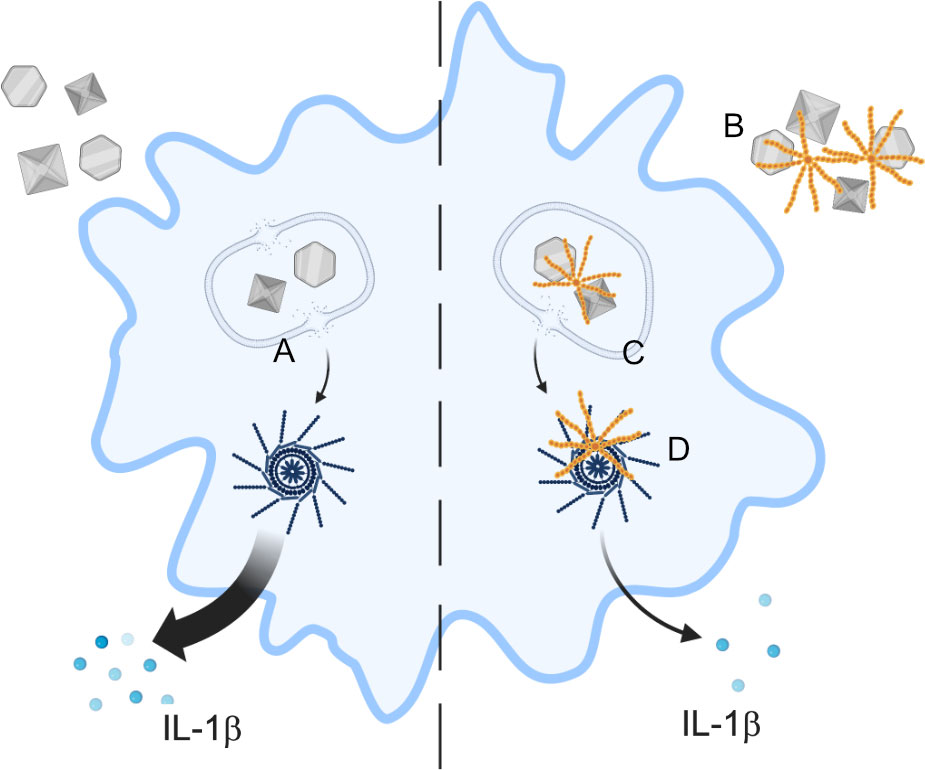
Proposed scheme of C4BP function. In absence of C4BP, particulates are taken up by macrophages and cause damage to the lysosomal membrane **(A)**, leading to content leakage and activation of the NLRP3 inflammasome, and IL-1β release. C4BP (orange) binds to extracellular particulates **(B)** and is co-internalised, limiting subsequent lysosomal membrane damage **(C)** and therefore inhibiting NLRP3 inflammasome activation. C4BP leaking from damaged lysosomes also associates with the inflammasome ASC speck **(D)**, but this does not appear to inhibit speck formation.

We previously identified that C4BP prevented IAPP fibrillation, and thereby preserved lysosomal membrane integrity. While MSU and SiO_2_ do not fibrillate or elongate, C4BP still protected against lysosomal membrane damage by MSU, as showed by increased MSU-induced galectin-3 puncta in the absence of C4BP (**Figure 6**). SiO_2_-induced galectin-3 puncta were not significantly reduced; however, we did identify a downwards trend in galectin-3 puncta when cells were treated with C4BP as compared to the BSA control condition. Given that C4BP strongly bound MSU and SiO_2_ (**Figures 1A, B**, **4D, E**) it is tempting to hypothesise that C4BP coating of the particulate inflammasome activator is sufficient to limit lysosomal membrane damage and resultant inflammasome activation (**Figure 8**). In this model, C4BP binding to the particulate would be crucial for NLRP3 inflammasome inhibition. As our *in vitro* experiments with HMDM were performed in the absence of serum, we can also conclude that the inflammasome-inhibitory activity of C4BP is independent of its complement inhibitory activity. Indeed, the complement regulatory C4b-binding region of C4BP α-chain is located within CCP domains 1 to 3 (21), whereas MSU/SiO_2_ binding is dependent on CCP domains 4 and 8 (**Figures 4D, E**).

Complement activation by particulates such as MSU leads to production of the anaphylotoxin C5a, which potentiates IL-1β release as well as causing direct complement membrane attack complex -dependent inflammasome activation (47). C4BP therefore likely limits inflammasome activation in the presence of serum at least partially due to its role as a complement inhibitor. In the *in vivo* model of IP MSU injection, cellular influx has been shown to be largely dependent on expression of ASC, caspase-1, and almost entirely dependent on presence of IL1R, demonstrating that it is predominantly inflammasome dependent (7). Our results from C4BP-knockout mice therefore support the role for inflammasome inhibition by C4BP *in vivo*. Our *in vitro* results in absence of serum also show a direct inhibitory effect of C4BP, independent of complement inhibition, at the stage of NLRP3 activation; C4BP alone did not affect inflammasome priming, as measured by pro-IL-1β expression, nor did it affect the general inflammatory potential of primed cells, as assessed by measuring secretion of IL-12 and IL-6 (13), demonstrating a mechanism of activity specific to the activation step of the NLRP3 inflammasome. By limiting both particulate-mediated complement activation, which can contribute to inflammasome priming and other inflammatory responses, as well as by directly limiting inflammasome activation, C4BP could therefore contribute to the “silent” uptake and clearance of insoluble particulates, in the same way as it is thought to contribute to silent clearance of apoptotic material (48).

Approximately 80% of circulating C4BP is bound to PS *via* the C4BP β-chain. Earlier studies have identified that PS mediates C4BP binding to apoptotic and necrotic cells due to interactions between PS and phosphatidylserine (49, 50). C4BP also binds necrotic cells *via* DNA exposed on the outer membrane of such cells, *via* its CCP1 and CCP2 domains, and limits pro-inflammatory signalling associated with necrosis (49). We found that recombinant human C4BP, which lacks the β-chain and is not bound to PS, potently inhibited SiO_2_- or MSU-induced inflammasome activation (**Figure 4**). Murine C4BP lacks the β-chain and was protective in an intraperitoneal *in vivo* model of MSU-induced inflammation (**Figure 7**) in male mice, confirming our HMDM data. Interestingly, female mice have significantly lower levels of serum C4BP, at only 60 μg/ml, compared to 160 μg/ml for male mice (39, 40), which is much closer to the reported human serum concentration of approximately 200 μg/ml (17). Accordingly, we saw a more significant effect of loss of C4BP in male compared to female mice (**Figure 7**). Overall, our data suggest that PS is not required for C4BP-mediated NLRP3 inflammasome inhibition. Additionally, we showed that, in contrast to cells undergoing another form of cell death, C4BP does not appear to have a strong affinity for cells undergoing pyroptosis, as shown by the lack of C4BP recovered from nigericin-stimulated HMDM (**Figures 2C, D**). Our data therefore provide further evidence that C4BP-mediated NLRP3 inflammasome inhibition depends on internalisation of C4BP and is not a consequence of C4BP binding to dying cells. Although particulates such as MSU, SiO_2_, and amyloid fibril formation all cause lysosomal membrane damage upstream of inflammasome activation, a common mechanism of NLRP3 activation for all diverse stimuli has been sought. One suggestion is the release of oxidised mitochondrial DNA into the cytosol (51). The binding of C4BP to DNA raises the possibility that C4BP internalised within the cell after uptake of particulates to which it is bound, could deliver C4BP to a site where it could interact with and neutralise oxidised mitochondrial DNA, as a mechanism of inhibition. This hypothesis would require future testing.

As well being a well-recognised complement inhibitor, C4BP has also been identified as having immunomodulatory activity on human monocyte-derived dendritic cells (Mo-DCs) (52), attributed to the C4BP α-chain CCP domain 6 (53). C4BP treatment of Mo-DCs induced a semi-mature, tolerogenic state, leading to downregulation of pro-inflammatory cytokine release, preventing activation marker upregulation, and inhibiting their ability to stimulate T-cell responses. We have now shown here that C4BP also inhibits particulate-stimulated NLRP3 inflammasome activation in HMDMs. The NLRP3 inflammasome is an extensively studied key component of the innate immune response that, when dysregulated, contributes to a large array of chronic and acute diseases. Often, onset and progression of such diseases is triggered by phagocytosis of insoluble protein aggregates, crystals or particles. Our laboratory has now established that C4BP inhibits NLRP3 inflammasome activation by IAPP, MSU crystals and SiO_2_ particles *in vitro*, suggesting C4BP may also inhibit NLRP3 inflammasome signalling by other disease-driving protein aggregates, crystals or particles. For example, NLRP3 inflammasome activation has been shown to be involved in Alzheimer’s disease (triggered by amyloid β aggregates (14, 54)), atherosclerosis (triggered by cholesterol crystals (55)) and asbestosis (triggered by asbestos particles (6)), and whether C4BP can inhibit NLRP3 inflammasome signalling by these activators should be formally investigated in future studies. The increased inflammatory phenotype of *C4bp* ^-/-^ mice upon intraperitoneal MSU injection suggests C4BP may be an interesting therapeutical target for treatment of inflammasome-driven diseases. Future investigations should also be aimed to identify whether (1); endogenous C4BP is protective by comparing inducible NLRP3 inflammasome-driven disease progression in WT and *C4bp* ^-/-^ mice (e.g. collagen-induced arthritis or silicosis), and (2) whether injected recombinant C4BP is protective in inflammasome-driven mouse disease models, such as the APP/PS1 Alzheimer’s disease model (56).

In summary, our study provides a better understanding into how C4BP inhibits crystal- or particle-induced NLRP3 inflammasome signalling. We propose that C4BP can directly bind such molecules, and once internalised, can perform its inflammasome inhibitory function by preventing lysosomal membrane damage (**Figure 8**). Our data thus suggest a novel mechanism by which the endogenously expressed C4BP protein mediates NLRP3 inflammasome signalling in human primary macrophages.

## Data availability statement

The raw data supporting the conclusions of this article will be made available by the authors, without undue reservation.

## Ethics statement

The studies involving human participants were reviewed and approved by the ethical committee for Lund University. The patients/participants provided their written informed consent to participate in this study. The animal study was reviewed and approved by the institutional animal experimentation committee of the Medical University of Vienna and the Austrian Ministry of Science.

## Author contributions

DB, NP-M, VK, and IB designed, executed, and analysed experiments. FM, SB, and MD provided C4BP mutants and recombinant C4BP used in the study. DB, SE, CB, AB, and BK contributed to experimental design and interpretation of data. DB, AB, and BK wrote and edited the manuscript with input from all authors. All authors contributed to the article and approved the submitted version.
