# Supplementary material for: C4b-binding protein inhibits particulate- and crystalline-induced NLRP3 inflammasome activation
Source: Front Immunol. 2023 May 22;14:1149822. doi: 10.3389/fimmu.2023.1149822 (PMC10239802; doi:10.3389/fimmu.2023.1149822)
Supplement: Supplementary file 1 [file Image_1.pdf]

Bierschenk et. al., supplementary figures:

Supplementary figure 1

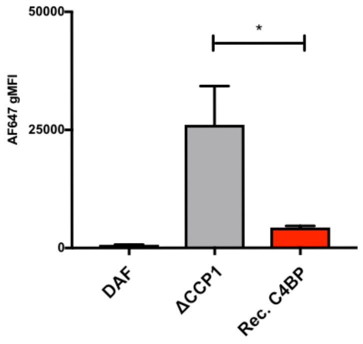

Supplementary Figure 1: C4BP CCP1 deletion enhances binding to MSU dramatically. Flowcytometric analysis of Alexa Fluor 647-labeled recombinant recombinant C4BP (Rec. C4BP), DAF, or the ΔCCP1 mutant. Bar graphs are mean+SEM of data pooled from 4 independent experiments. Statistical significane was tested by one-way ANOVA using the Dunnett's multiple comparison test. gMFI, geometric mean fluorescence intensity.

Supplementary figure 2

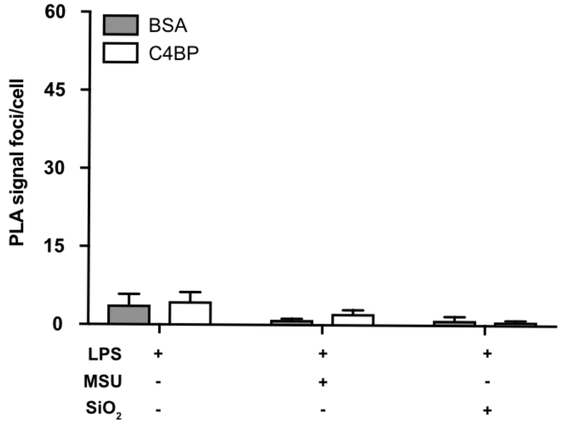

Supplementary Figure 2. IgG control antibodies show little PLA foci. Determination of C4BP-ASC PLA signal foci/cell in inflammasome-stimulated HMDM, where C4BP and ASC antibodies were substituted for IgG control antibodies. Bar graphs are mean + SEM of data pooled from 4 independent experiments.

Supplementary figure 3

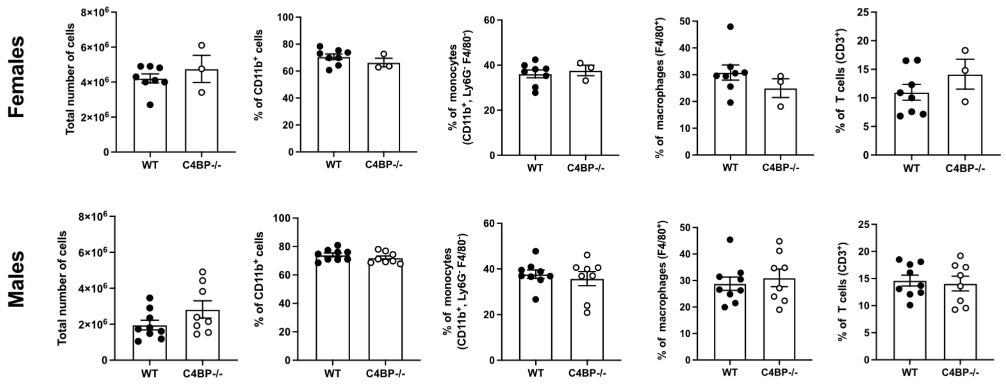

Supplementary Figure 3. Quantification of immune cell populations in peritoneal lavage fluid of untreated female (top row) and male (bottom row) C4BP-/- mice, and WT controls. No significant differences were seen in total number of cells, or in populations of total CD11b+ cells, macrophages, monocytes or T-cells. Neutrophils made up less than 0.4% of lavaged cells (not shown) and also showed no significant differences between WT or KO mice. Lack of C4BP therefore does not affect resident cell populations in untreated mice.
